# Supplementary material for: The Roles of Bacteria in Soil Organic Carbon Accumulation under Nitrogen Deposition in Stipa baicalensis Steppe
Source: Microorganisms. 2020 Feb 26;8(3):326. doi: 10.3390/microorganisms8030326 (PMC7142556; doi:10.3390/microorganisms8030326)
Supplement: Supplementary file 1 [file microorganisms-08-00326-s001.pdf]

microorganisms

Article

The Roles of Bacteria in Soil Organic Carbon Accumulation under Nitrogen Deposition in *Stipa baicalensis* Steppe

Jie Qin [1,†], Hongmei Liu [1,†], Jianning Zhao [1], Hui Wang [1], Haifang Zhang [1], Dianlin Yang [1,*] and Naiqing Zhang [2,*]

[1] Agro-Environmental Protection Institute, Ministry of Agriculture and Rural Affairs; Key Laboratory of Original Agro-Environmental Pollution Prevention and Control, MARA; Tianjin Key Laboratory of Agro-Environment and Agro-Product Safety, Tianjin 300191, China; qinjie@caas.cn (J.Q.); liuhongmei@caas.cn (H.L.); zhaojianning@caas.cn (J.Z.); wanghui03@caas.cn (H.W.); hfzhang12@126.com (H.Z.)

[2] Department of Ecology and Landscape Architecture, Dezhou University, Dezhou 253023, China

* Correspondence: yangdianlin@caas.cn (D.Y.); nqzh67@126.com (N.Z.); Tel.: +00-86-022-23611820 (D.Y.)

† These authors contributed equally to this paper.

Received: 8 December 2019; Accepted: 23 February 2020; Published: date

Supplemental Information

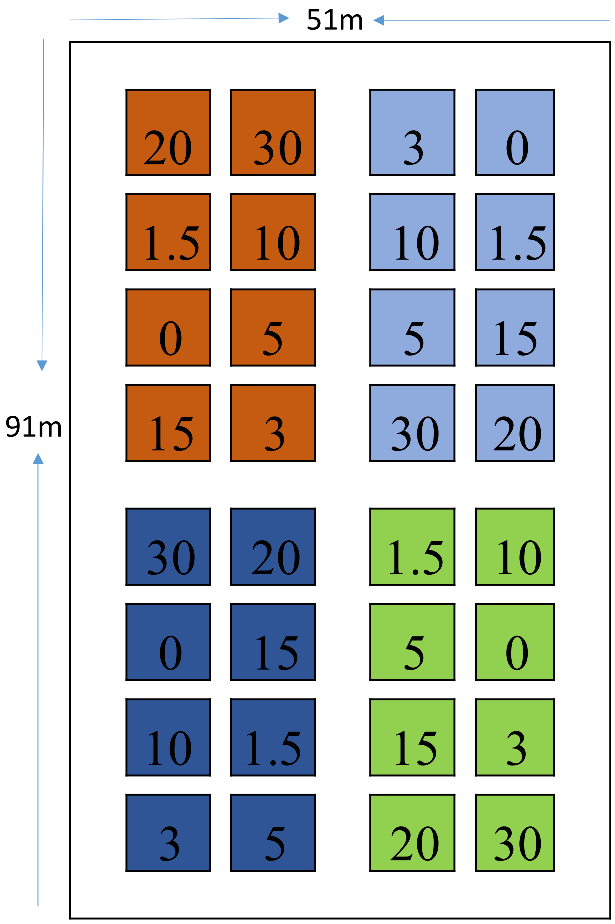

Figure S1. Schematic diagram of the N addition treatment. 0, 1.5, 3.0, 5.0, 10.0, 15.0, 20.0 and 30.0 g N·m^{-2} yr^{-1}

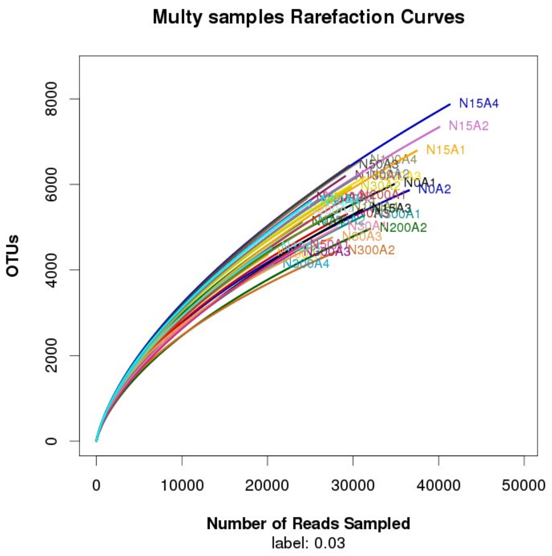

Figure S2. Multisample rarefaction curves

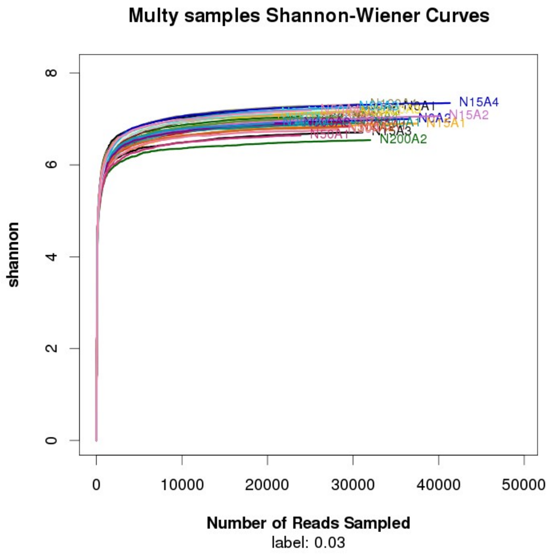

Figure S3. Multisample Shannon-Wiener curves

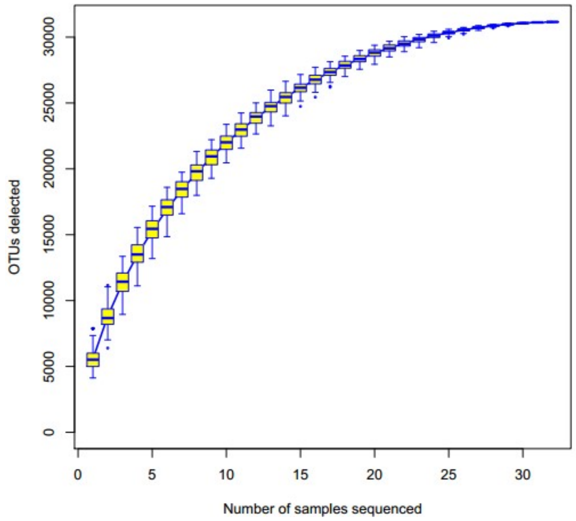

Figure S4. Species accumulation curve

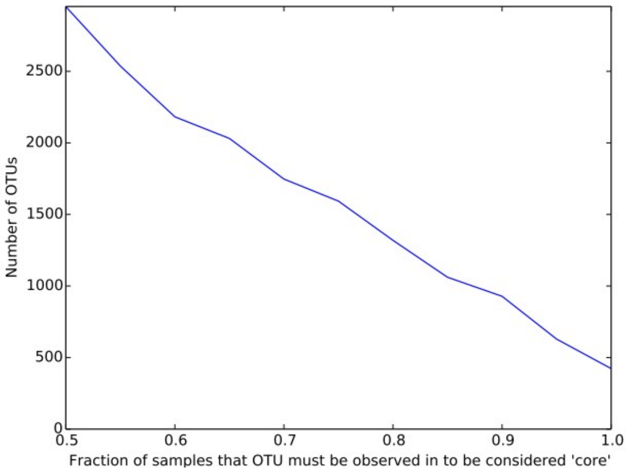

Figure S5. Relationship between sample rate and number of OTUs

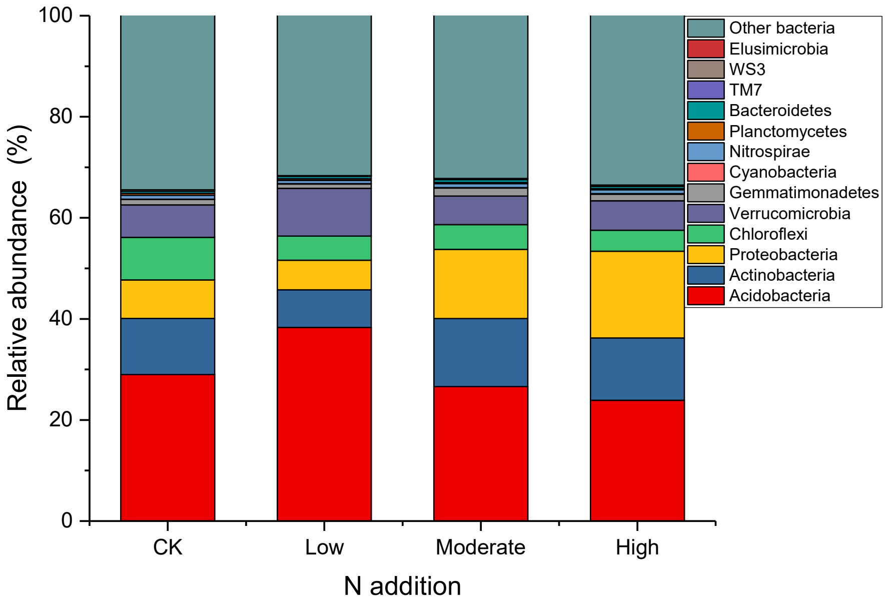

Figure S6. Effects of N addition on dominant bacterial phyla.

Table S1. Results (F values) of one-way ANOVAs of the effects of the nitrogen addition gradient on soil moisture, pH, NO_3-N, NH_4-N, total N, C:N, available P, total P, microbial biomass carbon, diversity and relative abundance of bacteria.

	F	*P*
Soil properties		
Total organic C	5.530	0.004
pH	11.386	0.000
NO_3-N	14.630	0.000
NH_4-N	10.556	0.000
Diversity of bacteria		
OTUs	4.857	0.008
chao1	1.109	0.362
goods_coverage	0.526	0.668
observed_species	1.123	0.356
PD_whole_tree	0.844	0.481
Shannon	1.407	0.262
Relative abundance of bacteria		
Acidobacteria	2.605	0.076
Actinobacteria	1.442	0.252
Proteobacteria	7.516	0.001
Cyanobacteria	0.077	0.972
Chlorofleri	2.679	0.066
Verrucomicrobia	2.096	0.123
Gemnatimonadetes	1.381	0.269
Nitrospirae	0.450	0.719
Planctomycetes	1.892	0.154
Bacteroidetes	0.816	0.496
TM7	2.139	0.057
WS3	4.677	0.118
Elusimicrobia	0.936	0.436

Statistical significance: **P*< 0.05, ***P*< 0.01, ****P*< 0.001.

Table S2. Pearson correlations between soil properties, bacterial diversity, relative abundance of bacterial phyla and total soil organic carbon

	r^2	*P*
Soil properties		
pH	-0.809**	0.000
NO_3-N	0.897**	0.000
NH_4-N	-0.119	0.516
Diversity of bacteria		
OTUs	-0.368*	0.038
chao1	0.314	0.081
goods_coverage	-0.172	0.346
observed_species	-0.404*	0.022
PD_whole_tree	-0.381*	0.032
Shannon	-0.195	0.284
Relative abundance of bacteria		
Acidobacteria	-0.282	0.154
Actinobacteria	0.059	0.749
Proteobacteria	0.344	0.079
Cyanobacteria	-0.198	0.277
Chlorofleri	-0.277	0.125
Verrucomicrobia	-.387*	0.029
Gemnatimonadetes	0.063	0.731
Nitrospirae	0.296	0.100
Planctomycetes	-.415*	0.018
Bacteroidetes	-0.028	0.879
TM7	0.149	0.415
WS3	0.312	0.082
Elusimicrobia	0.178	0.328

Statistical significance: *P< 0.05, **P< 0.01

Table S3. Results of structural equation modeling of N addition effects on soil organic carbon through all plausible interaction pathways. The table shows the unstandardized path coefficients (estimates), standard error of regression weight (S.E.), the critical value for the regression weight (C.R.), and level of significance for the regression weight (*P*). *** indicates $P \leq 0.001$, ** indicates $P \leq 0.01$, * indicates $P \leq 0.05$

Path			Estimate	S.E.	C.R.	*P*
pH	<---	N addition	-0.654	0.136	-4.813	***
NO_3-N	<---	N addition	0.702	0.128	5.489	***
Bacterial diversity	<---	pH	0.165	0.205	0.804	0.421
Proteobacteria	<---	pH	-0.412	0.193	-2.136	0.033
Planctomycetes	<---	pH	0.523	0.184	2.838	0.005
Bacterial diversity	<---	NO_3-N	0.108	0.205	0.529	0.597
Proteobacteria	<---	NO_3-N	-0.114	0.193	-0.592	0.554
Planctomycetes	<---	NO_3-N	0.186	0.184	1.011	0.312
TOC	<---	N addition	0.326	0.12	2.716	0.007
TOC	<---	Bacterial diversity	-0.145	0.123	-1.18	0.238
TOC	<---	Proteobacteria	0.349	0.128	2.722	0.006
TOC	<---	Planctomycetes	-0.35	0.133	-2.623	0.009
